# Inflammation triggered by the NLRP3 inflammasome is a critical driver of diabetic bladder dysfunction

**DOI:** 10.3389/fphys.2022.920487

**Published:** 2022-11-25

**Authors:** Francis M. Hughes, Michael R. Odom, Anissa Cervantes, J.Todd Purves

**Affiliations:** Division of Urology, Department of Surgery, Duke University Medical Center, Durham, NC, United States

**Keywords:** bladder, uropathy, cystopathy, inflammasome, inflammation, NLRP3, innate immunity

## Abstract

Diabetes is a rapidly expanding epidemic projected to affect as many as 1 in 3 Americans by 2050. This disease is characterized by devastating complications brought about high glucose and metabolic derangement. The most common of these complications is diabetic bladder dysfunction (DBD) and estimates suggest that 50–80% of patients experience this disorder. Unfortunately, the Epidemiology of Diabetes Interventions and Complications Study suggests that strict glucose control does not decrease ones risk for incontinence, although it does decrease the risk of other complications such as retinopathy, nephropathy and neuropathy. Thus, there is a significant unmet need to better understand DBD in order to develop targeted therapies to alleviate patient suffering. Recently, the research community has come to understand that diabetes produces a systemic state of low-level inflammation known as meta-inflammation and attention has focused on a role for the sterile inflammation-inducing structure known as the NLRP3 inflammasome. In this review, we will examine the evidence that NLRP3 plays a central role in inducing DBD and driving its progression towards an underactive phenotype.

## 1 Introduction and background

While early healers undoubtedly noticed that urinary abnormalities, particularly polyuria, were associated with diabetes, one of the first physicians to report on this was the notable Dr. William Prout, an early 19th century physician and, unusual for the time, chemist in England who had a penchant for studying urinary organs (Rosenfeld, 2003). Some of the earliest reporting by Dr. Prout on a connection between diabetes and bladder dysfunction dates back to the early-mid 1800s (Prout, 1843, 1849) By the late 1800s some clinicians were even reporting successful treatments to help alleviate this problem (Finlayson, 1881), although many credit Bowen and Aaron in 1926 as the first to make this distinction (Bowen and Aaron, 1926; Spring and Hymes, 1953). Known as diabetic bladder dysfunction (DBD), diabetic uropathy, or diabetic cystopathy, initial estimates of the prevalence of this complication were highly varied, ranging from 25 to 87% (Frimodt-Moller, 1980), One study of diabetic patients in Italy, which excluded patients with several confounders including neurogenic bladder or prior spinal or pelvic surgery, found that about 61% of their patients had urological symptoms (Bolgeo et al., 2020). Another study that excluded other potential causes, but only included female patients, found that 67% of their patients were experiencing lower urinary tract symptoms (LUTS) (Karoli et al., 2014). In diabetic children aged 11–17, the prevalence of LUTS was found to be 33.3%, which was twice as high as in children without diabetes (Kelly et al., 2018). Other studies examined the prevalence of overactive bladder, one of the manifestations of DBD, in patients with diabetes, and the prevalence ranged from 13.9% to 24.2% (Liu et al., 2011; Ikeda and Nozawa, 2015; Xu et al., 2017). Despite this variability, there is a general consensus that the incidence of DBD is around 50% (Arrellano-Valdez et al., 2014; Yuan et al., 2015). This places DBD as one of the most common diabetic complications, yet it has not received as much attention as some of the better-known complications such as retinopathy, neuropathy and nephropathy. Perhaps this is because DBD is not perceived to be as detrimental to one’s health or as morbid as other complications, but it is well known to have profound effects on the quality of life for these patients (Wittig et al., 2019)

## 2 Clinical presentation of diabetic bladder dysfunction

Diabetic bladder dysfunction (DBD) is an umbrella term for a wide variety of signs and symptoms related to urinary function in diabetic patients. It was classically described in the literature as a triad of decreased bladder sensation, increased bladder capacity, and impaired detrusor contractility (Frimodt-Moller, 1978). However, more modern reports include a variety of urodynamic findings in patients with diabetic bladder dysfunction rather than this classic triad. Another complicating factor is that the DBD often co-presents with other urological pathologies such as bladder outlet obstruction (BOO) (Kaplan et al., 1995). To eliminate some of these possible confounders, some studies have examined DBD only in women without a history of neurogenic bladder disorders. One such study compared urodynamic findings between diabetic and nondiabetic women who presented with similar urological complaints and found that diabetic women were more likely to have increased bladder capacities, detrusor underactivity, and impaired sensation at 75% of capacity (Malik et al., 2020), which resembles the classic triad first proposed. To conceptualize such conflicting and varied presentations, Daneshagari et al. proposed a temporal model (Daneshgari et al., 2009b). The early stage is marked by storage problems and is thought to be due to polyuria which leads to bladder compensation *via* hypertrophy. This compensated state may lead to detrusor muscle hypercontractility which presents as overactive bladder (OAB) symptoms including increased frequency, urgency and urge incontinence. In contrast, our more recent study in Akita diabetic mice found that inflammation caused by high glucose, not polyuria, triggered OAB symptoms (Inouye et al., 2018). Following OAB, a subsequent stage was recognized that reflects underactive bladder (UAB) and presents with increased void volumes, decreased voiding frequency, high post-void residual volumes (PVR), decompensation and, ultimately, overflow incontinence (Daneshgari et al., 2009b). This temporal model assumes that the duration and severity of diabetes will determine the presentation, but there is conflicting data around this assumption.

While positive correlations are often seen between urological complications and the duration/severity of diabetes, significant relationships are few (Tam et al., 2017; Majima et al., 2019; Papaefstathiou et al., 2019). One of the few significant relationships that appeared in multiple studies was a relationship between urinary incontinence and increased HgbA1c (Wang et al., 2015; Wessells et al., 2018). However, another study examining incontinence in Palestinian women with type 2 diabetes did not find a relationship between incontinence and the duration, treatment type, or complications of diabetes (Nazzal et al., 2021). Other studies have tried to draw correlations between other diabetic complications (e.g., nephropathy, neuropathy, retinopathy) and DBD symptoms with equally mixed results. Karoli et al. found that peripheral neuropathy, nephropathy, and metabolic syndrome had a significant positive odds ratio with lower urinary tract symptoms (LUTS) in women with type 2 diabetes (Karoli et al., 2014). In contrast, Majima et al. found in a multivariate analysis that only diabetic retinopathy was significantly correlated with bladder contractility index but neither diabetic retinopathy nor nephropathy was significantly associated with maximum cystometric capacity (Majima et al., 2019).

From a treatment perspective, diabetic bladder dysfunction stands out amongst other well-known diabetic complications, such as retinopathy and nephropathy, in that aggressive glycemic controls do not effectively decrease the severity of symptoms. The Epidemiology of Diabetes Interventions and Complications (EDIC) Study, the long-term observational follow-up of the Diabetes Control and Complications Trial (DCCT), found that many diabetic complications can be diminished *via* strictly controlling blood sugar (Genuth, 2006). However, in the subset of Type 1 diabetic men in the subsequent UroEDIC trial that focused on urinary dysfunction, no beneficial effect was seen in intensive *versus* conventional treatment protocols as measured by AUA symptom scores (Van Den Eeden et al., 2009). There are several possibilities for this observation, including the simple fact that most men in the study were younger than those normally seen with LUTs and so an insufficient number of subjects had developed measurable urinary symptoms during the observed time-period. A more intriguing possibility is that the effects of metabolic derangement, possibly inflammatory, produce a cascade of progressive consequences that include irreversible genetic, epigenetic, cellular and tissue changes, known collectively as “metabolic memory” (Ceriello et al., 2009; Copur et al., 2021; Zheng et al., 2021), that are no longer directly affected by glucose control. This phenomenon applies to many diabetic complications which are incompletely restored by glucose control, although DBD seems to be particularly recalcitrant in that regard. Patients frequently have metabolic dysregulation long before their diagnosis/treatment and chronic inflammation and metabolic memory could be established during this period. If this proves to be correct, intervention will require both a means to arrest the ongoing inflammatory process and a strategy to reverse the physical deterioration of bladder function.

The prevalence of DBD is elusive due to the difficulty in defining it, the frequency of concomitant urological disorders, and the variability of the population studied. Based on Daneshagari’s temporal model, modern theories of DBD (Daneshgari et al., 2006a; Daneshgari et al., 2006b; Daneshgari et al., 2009b) suggest that symptoms of bladder overactivity, such as increased urinary frequency and urgency, are the first symptoms of DBD to develop. These are then followed by UAB symptoms such as hesitancy, slow urinary stream, and bladder decompensation. While, in reality, patients may experience symptoms typical of one or more of these states (Gomez et al., 2011), there is evidence that progression may be occurring in humans (Kirschner-Hermanns et al., 2012). It is likely that the propensity for humans to avoid seeking medical care early, along with the relatively quiet onset of diabetes, may mask the clear progression in humans and result in the wide range of presenting symptoms. Clearly, definitive longitudinal studies are profoundly needed in humans. Equally needed are animal models that recapitulate this progression so that the mechanisms driving it can be studied in detail.

## 3 Animal models of diabetic bladder dysfunction that progress from overactive bladder to underactive bladder

### 3.1 Type 1 diabetes mellitus

#### 3.1.1 Streptozotocin model

There are numerous models of diabetes and urinary function has only been investigated in a handful of these. The first indication of a progression from OAB to UAB in an animal model was discovered in an often-used model of type 1 diabetes mellitus (T1DM), the streptozotocin-induced model. Streptozotocin (STZ) is an antibiotic derived from *Streptomyces* achromogenes that has been used to induce a diabetic state in rodents and is the most widely published diabetic rodent model (Herr et al., 1959; Kolb, 1987; Pandey and Dvorakova, 2020). It is a cytotoxic glucose analogue and enters cells *via* glucose transporter 2 (GLUT2) making it particularly toxic to pancreatic islet beta cells (Lenzen, 2008). STZ administration typically is given at a single high dose (inducing a T1DM model) or as multiple lower doses (resembling a T2DM model) (Wu and Yan, 2015). Increased water intake and urination is seen immediately after STZ induction, and decreased body weight and increased bladder weight becomes more severe over time (Christ et al., 2006; Leiria et al., 2011; Lee et al., 2018; Yang et al., 2019a). Additionally, bladder hypertrophy was present as soon as 1 week post induction and was similar across male and female rodents and across STZ doses (Arioglu Inan et al., 2018). Corresponding cystometric changes can be seen almost immediately but vary depending on the length of time that has passed since induction. Yang et al. showed STZ administered in male mice initially created an overactive bladder phase that peaked at 6 weeks and then transitioned to an underactive bladder state between 9 and 12 weeks (Yang et al., 2019a). An *ex vivo* study agreed with this finding by showing enhanced ATP release and nerve-evoked contractions at 2 weeks post STZ induction but decreased ATP release and contractility at 12 weeks (Lee et al., 2018). This is consistent with the temporal model proposed by Daneshgari et al. (Daneshgari et al., 2009a).

Insulin therapy was shown to only partially reverse bladder hypertrophy and cystometric changes when administration began shortly after induction or when it was administered weeks after induction (Christ et al., 2006; Arioglu Inan et al., 2018). A major limitation of this model is that sensitivity to STZ can be highly variable due to strain differences, gender differences, diet, time of day administered, administration route, and other factors (Deeds et al., 2011). For example, female mice typically require higher doses than males in order to achieve the same severity of diabetes (Saadane et al., 2020). In addition, there are major differences between mice and rats. On the other hand, a major benefit of this model is that the severity of diabetes can be established by the amount of STZ given (Junod et al., 1969).

#### 3.1.2 The Akita model

The Akita mouse arose from a spontaneous mutation in the *ins2* gene that causes it to fold incorrectly and produce toxicity in pancreatic β cells (Korkmaz et al., 2007; Kong et al., 2013). The result is a decreased β cell mass and reduced insulin production leading to increased blood sugar. The increase is usually apparent soon after weaning. Homozygote mutants have a very severe phenotype and suffer from premature death (usually in the perinatal period (Kong et al., 2013)) so studies are typically performed with heterozygotes. Males have a more severe phenotype than females although both have hyperglycemia, albuminuria and high blood pressure. These mice are used frequently in studies of diabetic nephropathy, (Gurley et al., 2006; Gurley et al., 2010; Kong et al., 2013), have been used in prior studies of DBD (Dolber et al., 2013), and were subsequently adapted by our lab to probe for changes in bladder function that would indicate DBD was progressing from OAB to UAB. We postulated that the less severe disease in the females may bring about a slower onset of the symptoms of DBD and, consequently, we may be better able to differentiate the OAB stage from the UAB. Thus, we began with females and found that by 15 weeks of age clear signs indicative of OAB were present during urodynamics, most tellingly an increase in urinary frequency coupled with a decrease in average void volume (Hughes et al., 2019a). Of course, in humans the main symptoms of OAB is frequency and urgency but urgency is impossible to quantify in rodents. Extending our studies in time revealed that by 30 weeks of age this urinary pattern had completely reversed itself with a decrease in frequency and a very pronounced increase in void volume (Hughes et al., 2022a), both of which are clear signs of UAB. Thus, it appears DBD in the Akita diabetic mouse progresses from OAB to UAB and should be an excellent model to study the progression of DBD.

It should be noted that most studies of diabetes in the Akita mice use the more severe male model and prior to our work one study had examined DBD in the male Akita (Dolber et al., 2013). Dolber et al. (Dolber et al., 2013) was studying the potential therapeutic value of angiotensin infusion on DBD and found evidence of UAB at 20 weeks of age in the male. Since this is 5 weeks after the females developed OAB, it is possible the males will have a stage of OAB similar to the females but, given the more severe diabetes, the progression is accelerated. Studies are currently underway to determine if these male mice have an OAB stage and thus demonstrate DBD progression.

### 3.2 Type II diabetes

#### 3.2.1 The high fat diet/low dose streptozotocin model

Since over 90% of all diabetic patients suffer from type II diabetes (T2DM), animal models which recapitulate this condition are in great demand. A key pathology in the development of T2DM is obesity. Fat quantity and type regulates inflammatory processes responsible for the development of insulin resistance, a hallmark finding in T2DM patients (Flanagan et al., 2008). A high fat diet fed to rats increases hepatic pro-inflammatory TNFα gene expression which leads to a loss of pancreatic function and subsequent insulin resistance (Flanagan et al., 2008). A HFD alone does not induce stark hyperglycemia but the pancreatic beta cell damage responsible for hyperglycemia and dysregulated glucose metabolism can be achieved through STZ injections. High doses of STZ cause extensive and rapid beta cell death resulting in the T1DM model discussed above. However, multiple low doses of STZ cause gradual beta cell death more consistent with the progression of human T2DM (Zhang et al., 2008). Rats that are fed a high fat diet and receive multiple low doses of STZ (30 mg/kg i.p. every 2 weeks) develop the constellation of symptoms consistent with T2DM such as an increase in body weight, insulin resistance, hyperglycemia, and blood lipid disorder (Zhang et al., 2008; Zhang et al., 2012a; Li et al., 2018). This diabetic model also demonstrates a high level of systemic inflammation. Circulating pro-inflammatory cytokines IL-6, TNFα, and MMP9 are upregulated in HFD/STZ induced diabetic rats (Peng et al., 2016). More importantly, this model develops progressively worsening DBD. After 1 month of HFD/STZ treatment, males develop an overactive detrusor characterized by an increase in voiding frequency and decrease in voiding volume (Lee et al., 2021). However, 4 months after induction, diabetic rats exhibit a significant decrease in voiding frequency, increase in void volume and an increased post void residual volume consistent with an underactive detrusor phenotype (Zhang et al., 2012a; Klee et al., 2019). Therefore, this inducible T2DM model progresses from an overactive to underactive detrusor phenotype and should be useful for examining the role of bladder inflammation in triggering this progression. Unfortunately, studies to date have yet to explore this connection, despite it being commonly used for well over a decade (Zhang et al., 2008; Zhang et al., 2012b; Li et al., 2018; Yesilyurt et al., 2019).

#### 3.2.2 Double knock out of insulin receptor substrate 1 and 2

An intriguing genetic mouse model of T2DM, which develops both detrusor overactivity and underactivity, has been developed through a conditional knock out of hepatic insulin receptor substrate 1 and 2 genes (Wang et al., 2012). This causes female mice (male mice were not examined) to develop diabetes and subsequent bladder dysfunction. Between the ages of 6–12 weeks, these diabetic mice develop overactivity defined by an increase in non-voiding contractions and a decrease in voiding volume. Conversely, underactivity develops between the ages of 16–20 weeks. At both time points, an increase in serum TNFα was observed. Such systemic inflammation is commonly associated with diabetes, but, interestingly, TNFα was also increased in bladder smooth muscle. The authors further demonstrated, somewhat surprisingly, that the pro-inflammatory cytokine TNFα directly promotes bladder smooth muscle contraction *in vitro*. Inhibiting TNFα activity using soluble TNF receptor 1 *in vivo* reduced TNFα levels and successfully improved bladder function in DKO mice.

### 3.3 Diabetic bladder dysfunction is associated with inflammation—Human studies

Decades of research have provided considerable insight into how the aberrant metabolism of diabetes leads to end organ damage and functional deficits. This was originally summarized in Brownlee’s unifying theory of diabetic complications (Brownlee, 2005). Briefly, hyperglycemia produces several contributory changes including 1) an increase in reactive oxygen species (ROS) production from the elevated metabolism, 2) an increase in the polyol and hexosamine pathway flux, 3) formation of advanced glycated end products (AGEs) and 4) activation of the protein kinase C pathway (Brownlee, 2005). Although not appreciated at the time Brownlee published his theory, it is now known that metabolites from these aberrantly regulated pathways activate inflammation (Shin et al., 2015) and that this inflammation is responsible for, or at least a major contributor to, many of the diabetic complications. For example, chronic inflammation and related stresses are associated with diabetic complications such as atherosclerosis, neuropathy, retinopathy, nephropathy, cardiomyopathy, erectile dysfunction and DBD (Wang and Kuo, 2017; Maiorino et al., 2018; Wang et al., 2020a; Baum et al., 2021; Munoz-Cordova et al., 2021; Sun and Ding, 2021; Lima et al., 2022; Rohm et al., 2022).

Although most studies of inflammation and DBD are in animals, at least one seminal study has been performed in humans (Wang and Kuo, 2017; Wang et al., 2020a). Wang et al. (Wang and Kuo, 2017) examined bladder biopsies from 19 patients with OAB and diabetes (and 10 healthy control patients) and found numerous markers of chronic inflammation, such as increased mast cell numbers and apoptosis along with decreased tight junction protein levels. Interestingly, he saw similar inflammatory changes in patients with OAB but an unknown etiology, suggesting that inflammation in the bladder, from whatever source, triggers OAB. Thus, there is reason to believe that investigations into the role of inflammation in DBD in animal models should be translatable to humans.

### 3.4 NLRP3-induced inflammation is a central driver of diabetic bladder dysfunction

#### Introduction to the innate immune system and inflammasome

The idea that inflammation plays a precipitating and/or exacerbating role in diabetic complications in general, and DBD specifically, is not controversial as the evidence appears overwhelming. Since the inflammation is driven by metabolic dysregulation and not by an invading pathogen, it is considered to be of a sterile form in end organs. The bladder is somewhat more complicated in the sense that urinary tract infections are more common in diabetic patients. However, DBD develops even in the absence of infection, suggesting that the contribution of pathogens to DBD is secondary to sterile inflammation. Indeed, it seems likely the sterile inflammatory damage to the barrier function of the urothelia leads to increased invasion of bacteria and thus the increased UTI rate.

Our understanding of the mechanism that brings about sterile inflammation was advanced significantly in 2002 when Tschopp et al. first discovered a structure they named the inflammasome (Martinon et al., 2002). The unique aspect of the inflammasome is that it was able to sense diverse stress signals and convert that into a conventional pro-inflammatory signal. The stress signals became known as danger- (or sometimes damage-) associated molecular patterns or DAMPS. These molecules are released from damaged or dying cells but can also be specific metabolites, which is likely a critical distinction with diabetes (Takeuchi and Akira, 2010; Wang et al., 2020b; Zhou et al., 2020). There are numerous DAMPs but some are particularly relevant to diabetes (Shin et al., 2015) such as monosodium urate (MSU), high-mobility group box 1 (HMGB1), C6-ceramide and AGEs. A similar pattern recognition concept exists with pathogenic molecules, known as pathogen associated molecular patterns or PAMPS. DAMPS and PAMPS are recognized by Pattern Recognition Receptors (PPRs) expressed by many different cell types including fibroblasts, myeloid immune cells, and epithelial cells (Lamkanfi and Dixit, 2011). There are five different families of PPRs: toll-like receptors (TLRs), C-type lectin receptors, retinoic acid-inducible gene-I (RIG-1)-like receptors, Absent in Melanoma 2 (Aim2)-like receptors, and nucleotide-binding oligomerization domain-like receptors or Nod-like receptors (NLRs) (Takeuchi and Akira, 2010). Of all these PPRs, only some of the NLRs and two of the AIM2-like receptors actually form inflammasomes. By far the best studied is the NLRP3 inflammasome which is believed to mediate the vast majority of sterile inflammatory responses in the body.

### 3.5 The NLRP3 inflammasome

**FIGURE 1:**
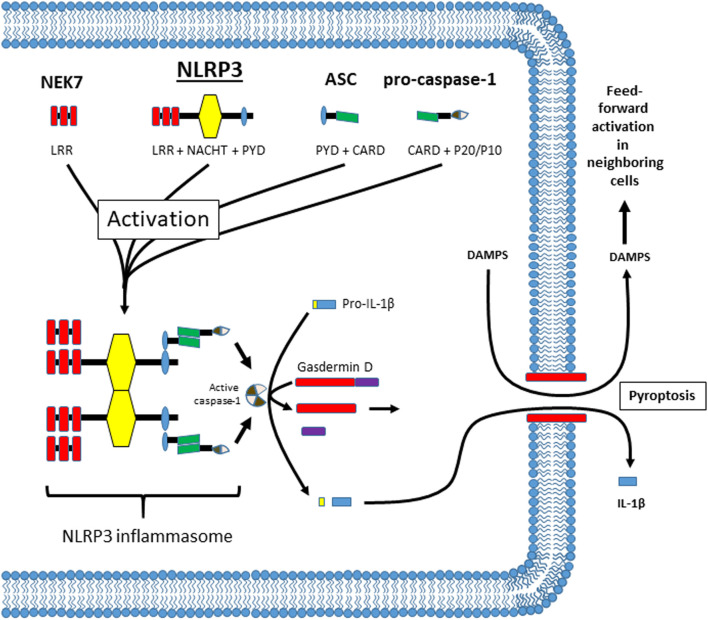
Illustration of the components of the NLRP3 inflammasome, its oligomerization after activation, and activities thereafter. The components of the NLRP3 inflammasome are illustrated at the top and are NEK7, NLRP3, ASC and pro-caspase-1. The individual domains in each protein are illustrated and named below the illustration (.i.e. LRR, NACHT, PYD, etc.). The exact mechanism of activation is highly debated but may take place through several different pathways. The result is that NEK7 facilitates NLRP3 oligomerization through the NACHT domains on NLRP3. In this figure only 2 individual NLRP3 molecules are shown for simplicity. ASC then binds NLRP3 through homotypic binding of its PYD domain. The CARD domain on ASC then binds the CARD domain on pro-caspase-1 and caspase-1 is cleaved through induced proximity. Cleavage releases a P20 and a P10 subunit which combines with the same subunits from the cleavage of another molecule of pro-caspase-1 to form an active tetramer. Active caspase-1 then cleaves pro-IL-1β and gasdermin D. It also cleaves pro-IL-18 if it is present but this cleavage is not shown for simplicity. The n-terminus of gasdermin D then moves to the plasma membrane where it assembles into a pore. This pore then facilitates a programmed necrosis termed pyroptosis. By lysing the cells, pyroptosis releases the mature IL-1β while also releasing intracellular DAMPs (and all the intracellular components) which then can activate NLRP3 in nearby cells in a feed forward fashion.

The NLRP3 inflammasome has been implicated in a wide variety of inflammatory pathologies, including most, if not all, diabetic complications (Yu et al., 2020; Bai et al., 2021; Lu et al., 2021; Mamun et al., 2021). As shown in Figure 1, NLRP3 is a member of the NLR family and normally exists as a soluble protein in the cytoplasm. It has 3 main domains: a C-terminal leucine-rich repeat (LRR) domain that senses ligand (probably indirectly), a nucleotide-binding and oligomerization (NACHT) domain that mediates ATP-dependent oligomerization, and an N-terminal pyrin (PYD) domain that mediates homotypic binding. The mechanism by which cytoplasmic NLRP3 senses extracellular DAMPS is often hotly debated but may consist of K^+^ efflux, reactive oxygen species (ROS) generation, Ca^2+^ flux, disruption of lysosomal membranes, or some combination of these signals (Vanaja et al., 2015; Malik and Kanneganti, 2017; Swanson et al., 2019; Yang et al., 2019b; Mamun et al., 2021). In addition, there are several ways NLRP3 can be activated after ligand recognition; known as the canonical, non-canonical and alternative pathways. The details of these pathways are beyond the scope of this report (for review see references (Pellegrini et al., 2017; Mathur et al., 2018; Downs et al., 2020; Xiang et al., 2020; Yi, 2020; Moretti and Blander, 2021; Yi, 2021)). In general however, activation of NLRP3 begins when the LRR domain of Nek7 (a NIMA-related kinase family member) (He et al., 2016; Shi et al., 2016) interacts with the LRR domain of NLRP3 causing the NLRP3 to oligomerize through binding of NACHT domains. As it comes together, the PYD domains bind to the same domain on an adaptor known as the apoptosis-associated speck-like protein containing a CARD (ASC). ASC consists of a PYD and a caspase recruitment domain (CARD) and the homotypic binding of the PYD results in the formation of long filaments (up to 1 µm) known as ASC specks. The CARD domain of ASC is a homophilic binding domain that binds to a CARD domain on the pro-protease known as pro-caspase-1.When pro-caspase-1 molecules are held in close proximity they undergo an autocatalytic cleavage process known as induced proximity that cleaves caspase-1 into p20 and p10 subunits. These subunits bind another identical set of subunits to form an active tetramer. The active caspase in turn cleaves pro-IL-1β and pro-IL-18 into their active forms (IL-1β and IL-18). In Figure 1, only IL-1β is shown for simplicity sake. To effectuate release of these cytokines, caspase-1 cleaves an additional protein known as gasdermin D (Shi et al., 2015). The N-terminus of gasdermin D then forms a pore in the cell membrane, facilitating a programmed form of necrosis known as pyroptosis (Aglietti et al., 2016; Ding et al., 2016; Sborgi et al., 2016). Pyroptosis allows the release of IL-1β (and IL-18) which then act as pro-inflammatory cytokines to initiate an inflammatory response. Pyroptosis also releases many intracellular DAMPS that can facilitate the activation of NLRP3 in neighboring cells, thus triggering further inflammation in a dangerous feed-forward mechanism.

### 3.6 NLRP3 in the bladder: Expression and implication in other diseases

Several studies have demonstrated the presence of NLRP3 (and other inflammasome-forming PPRs) in the bladder (Hughes et al., 2014; Hughes et al., 2015; Hughes et al., 2016a; Hughes et al., 2019a; Chen et al., 2019; Tudrej et al., 2019; Verma et al., 2019). In those studies, NLRP3 was expressed primarily in the urothelia with little or no expression in the interstitium or detrusor. This is perhaps not surprising since one of the main purposes of the urothelia is to serve as a barrier to toxins and bacteria in the urine and so, in the event of a breach in the barrier, NLRP3 in urothelia would be optimally positioned to initiate an inflammatory response. Recent studies have begun to explore a possible role of NLRP3 in a number of different inflammatory bladder pathologies. The NLRP3 inflammasome was first implicated in bladder inflammation in a cyclophosphamide-induced hemorrhagic cystitis model (Hughes et al., 2014) but, since that time, it has been implicated in other pathologies including bladder outlet obstruction (Hughes et al., 2016a; Lutolf et al., 2018; Chen et al., 2021), urinary tract infections (Hughes et al., 2016b; Demirel et al., 2018; Lindblad et al., 2019; Mao et al., 2021), chemotherapy (Hughes et al., 2014), stone damage (Harper et al., 2019), interstitial cystitis/painful bladder syndrome (Tudrej et al., 2019; Shih et al., 2021; Wang et al., 2021), cigarette smoke-induced bladder dysfunction (Wu et al., 2020; Ma et al., 2021), ageing (Chen et al., 2019) and, the focus of this review, diabetes (Hughes et al., 2019a; Hughes et al., 2022a; Odom et al., 2022).

DBD is a slow-onset and slow-progressing complication, yet release of IL-1β and IL-18 are commonly thought to trigger an acute response with classic inflammatory symptoms (fever, pain, swelling, etc.). However, when released at lower levels over extended periods of time, they lead to a chronic state of low-grade inflammation called meta-inflammation (Hotamisligil, 2006; Boni-Schnetzler and Meier, 2019; Russo et al., 2021) that does not result in such overt symptoms. Work from our lab has recently demonstrated this meta-inflammatory response in the bladder during diabetes (Hughes et al., 2019a).

### 3.7 Diabetic DAMPS activate NLRP3 in urothelia

Diabetic metabolites have been shown to trigger activation of the NLRP3 inflammasome in numerous diabetic complications. In the case of the bladder, exposure to these metabolic DAMPs certainly comes from exposure to systemic metabolites in circulation, as it does for all other tissues. However, the bladder is also unique for many of these metabolites are concentrated and stored in the urine prior to excretion. Normally the barrier function of the bladder, discussed more in depth below, would protect the interior bladder cells from these concentrated metabolites. However, activation of pyroptosis in the urothelial layers by systemic DAMPS can reduce the integrity of this barrier, allowing greater exposure to the concentrated urinary metabolites. To determine if these metabolites might directly activate NLRP3 in urothelia, we treated urothelial cells in culture with the various metabolites and measured the resulting caspase activity; the enzymatic product of inflammasome activation. We found that at least four diabetic DAMPS; MSU, HMGB-1, C6-ceramide, and AGEs all triggered inflammasome activation in a dose-dependent manner (Hughes et al., 2019a), supporting the hypothesis that these metabolites initiate and propagate the inflammation that leads to DBD. Particularly intriguing are the AGEs as non-enzymatic glycation of proteins and lipids is proposed to contribute to, or be responsible for, the “metabolic memory” that makes treatment of DBD recalcitrant to glucose control.

### 3.8 Role of NLRP3 in DBD

To investigate NLRP3 in DBD, we have used the Akita type 1 diabetic mouse model, discussed above. To investigate a role for NLRP3-induced inflammation these mice were crossbred to commercially available NLRP3 null mice (NLRP3^−/−^; Jackson Laboratory, Bar harbor, MA), to create 4 separate genetic groups.

1. Wild type, wild type (WT, WT) - these animals possess both wildtype INS2 genes and both wild type NLRP3 genes. They are simply the wildtype C57/blk6 mice.
2. Diabetic, wild type (Diab, WT)—these mice are heterozygote at the INS2 loci for the Akita mutation but also possess both wild type NLRP3 genes. They are the diabetic mice.
3. Wild type, NLRP3 knock out (WT, NLRP3^−/−^)—these are the commercially available NLRP3^−/−^ mice which are not diabetic. They are the knock out control.
4. Diabetic, NLRP3^−/−^ (Diab, NLRP3^−/−^)—these mice were bred specially for these studies. They are heterozygote at the INS2 loci for the Akita mutation (and thus are diabetic) while having NLRP3 genetically deleted.

#### 3.8.1 Inflammasome activation and inflammation during DBD

All the mice used were female to take advantage of the progression from OAB (at 15 weeks) to UAB (at 30 weeks), thus allowing analysis at two opposite extremes of urinary dysfunction. Blood glucose was not affected by the presence or absence of NLRP3. It was noted that caspase-1 (the enzymatic readout of NLRP3) was indeed active in the urothelia at 15 weeks suggesting the inflammatory pathway was activated. Next, all groups were assessed for inflammation using the Evans blue assay. This assay measures the increase in capillary leakage that occurs as part of a typical inflammatory response and allows, among other things, increased leukocyte extravasation into the tissue. Evans blue dye is injected intravenously where it is quickly distributed systemically. It then “leaks” across the capillary walls at a rate that correlates with severity of inflammation and can be measured in the extravascular space of the tissue. Using this assay we have shown diabetes in the Akita mouse causes a tremendous increase in inflammation in the bladder at 15 weeks (Hughes et al., 2019a). This dramatic increase was also apparent at 30 weeks (Hughes et al., 2022a), clearly showing that a persistent state of inflammation is evident in the bladder in the diabetics. Importantly, the genetic deletion of NLRP3 completely prevented this inflammation, highlighting the critical role of this inflammasome in the immune response to metabolic dysregulation.

#### 3.8.2 Diabetic bladder inflammation is driven by hyperglycemia, not polyuria

Beyond hyperglycemia-induced inflammation, diabetes also stresses the bladder by generating an osmotic diuresis that increases the urinary volume and thereby the work load of the bladder. Therefore, the inflammation in the bladder during DBD could theoretically be driven by either the high glucose (and the resulting aberrant metabolites), or by overuse of the bladder (polyuria). In a recent study we compared these diabetic Akita mice with Akita mice given Phlorizin (Inouye et al., 2018). Phlorizin is an inhibitor of sodium-glucose linked transporter types 1 and 2 that prevents glucose reabsorption in the kidney. The result is even greater polyuria but normalized blood glucose. In that study Phlorizin completely blocked bladder inflammation despite increasing urine output, clearly demonstrating that hyperglycemia and not polyuria is responsible for triggering bladder inflammation.

#### 3.8.3 DBD is mediated by NLRP3 activation

Urodynamics is the gold standard for assessing bladder function in both animal models and human patients and is, thus, a critical tool for investigating DBD. In the diabetic/NLRP3^+/+^ mice at 15 weeks, OAB was apparent from decreased voiding volumes and increased voiding frequency. This was accompanied by an increase in post void residual (the amount of urine remaining in the bladder after a void) and an overall decrease in voiding efficiency. Importantly, the pathological changes in all these parameters were prevented in the diabetic/NLRP3^−/−^ mice, clearly showing a critical role for NLRP3 in triggering OAB (Hughes et al., 2019a). In addition, we measured urinary function at 30 weeks, corresponding to middle age in mice (Hughes et al., 2022a). By this time, the persistent diabetes and inflammation had caused void volumes to increase dramatically, along with a decrease in frequency, suggesting a progression to an underactive bladder. Again, these changes were not present in the diabetic/NLRP3^−/−^ who showed void volumes and urinary frequency similar to the wild type mice. Thus, the progression to this latter-stage UAB is also dependent on NLRP3.

#### 3.8.4 Diabetic bladder denervation is driven by NLRP3

Peripheral neuropathy is a seminal diabetic complication that is thought to be responsible for, or contributory to, the various symptomologies of DBD (Deli et al., 2013; Daneshgari et al., 2017; Agochukwu-Mmonu et al., 2020). While neuropathy outside the bladder itself may certainly contribute to bladder dysfunction, as might parameters such as speed of neuronal signaling, there is an observable alteration in nerve numbers or nerve densities within the bladder itself. Recently, the overall bladder nerve numbers (i.e. PGP9.5^+^ neurons) were assessed in the Akita diabetic mouse at the OAB time point and both nerve numbers and nerve density were found to be reduced (Hughes et al., 2019a). Excitingly, neuronal loss did not occur in diabetic animals lacking a functional copy of the NLRP3 gene. While the mechanism of neuronal loss was not investigated, other studies of denervation in the bladder have shown that IL-1β, the cytokine output of the inflammasome, has direct apoptotic effects on nerves (Lutolf et al., 2018). Moreover, Anakinra, a FDA-approved recombinant version of the naturally occurring Interleukin-1 receptor antagonist, also blocked neuronal loss *in vivo* in those studies (Lutolf et al., 2018). So, while direct effects of IL-1β on neuronal apoptosis have yet to be explored in DBD (and the Akita model specifically), it is tempting to speculate this mechanism is also operational in this complication as both involve a central activation of NLRP3.

In addition to overall neuropathy, NLRP3-dependent changes in bladder nerves may explain specific urinary symptoms experienced by diabetics. For example, Aδ-fibers are known to relay a sense of bladder fullness to the central nervous system and diabetic patients often report a reduction in this sensation. Using the same set of Akita mice discussed above, at the OAB time point, the diabetic mice were found to have a decreased density of Aδ-fibers in the bladder wall. Importantly, this decrease was blocked in the NLRP3^−/−^ mice showing a major role for NLRP3 but also suggesting IL-1β-induced apoptosis may be the underlying mechanism. In contrast, C-fibers in the urothelium were actually increased in the diabetic mice in the compensated phase. C-fibers normally sense pain in the body but in the bladder they are also thought to help trigger the OAB phenotype (Fowler, 2002). Therefore, an increased population of these nerves may help explain the OAB symptoms at 15 weeks. The results certainly suggest IL-1β is not having direct apoptotic effects on C-fibers but, in contrast, is either stimulating proliferation or increasing outgrowth and branching (the experiments cannot differentiate between these two possibilities, which are not mutually exclusive). IL-1β has been shown to promote such neuronal outgrowth in other nerve types (Park et al., 2018). Finally, these studies have not yet been reported at the 30 weeks, UAB time point.

#### 3.8.5 Breakdown of the urothelial barrier and remodeling during DBD

A key function of bladder urothelia is to serve as a permeability barrier to protect underlying cells from noxious waste products, toxins, bacteria, and pro-inflammatory metabolites that accumulate in urine. Three distinct layers make up the urothelial lining: basal, intermediate, and apical or superficial cells (Khandelwal et al., 2009). While the basal and intermediate layers play important sensory functions, the barrier function is primarily exercised by the superficial cells. In the very luminal surface of these superficial cells the bladder has a layer of protection found solely in the urinary tract, which is likely indicative of the importance of this barrier. This layer is composed of individual proteins called uroplakins that form a hexagonal array or plaque on the surface and are very effective in preventing urine from penetrating into the tissue (Wu et al., 2009; Matuszewski et al., 2016). Below this unique barrier are important barrier junctions found in other epithelia; tight junctions and adherens junctions. Tight junctions are the most important of these as their proteins form water and ion-tight seals between cells and prevent unwanted paracellular transport of molecules (Hartsock and Nelson, 2008). Adherens junctions stabilize cell-cell adhesion and are physically linked with the tight junctions *via* zona occludens proteins (Campbell et al., 2017), thus they play important roles in stabilizing the barrier.

While studies of the response of epithelial barriers to diabetes are sparse (Gekka et al., 2004; Xia and Rizzolo, 2017; Riedel et al., 2021), one previous study did examine ultrastructural changes of the urothelia over time in the rat streptozotocin model (T1DM) (Hanna-Mitchell et al., 2013). Using electron microscopy, the study showed a loss of uroplakins, broken tight junctions and the lack of a clear distinction between apical and intermediate urothelial cell layers occurred after 9 weeks. By 20 weeks, the urothelial layer was restored, albeit with an altered phenotype. Unfortunately, they did not actually measure permeability nor inflammation. In a recent study (Odom et al., 2022), we employed the female Akita model discussed above and examined if the bladder permeability is altered during diabetes, if this changes over time and if there was a role for NLRP3-induced inflammation. For this investigation, we examined bladders during both the OAB and UAB state, rationalizing permeability may be different between these two groups. We found that mice exhibiting OAB had a profound increase in urothelia barrier permeability which was evident by movement of small molecules (Evans blue dye) across urothelial layers that had been removed of their detrusor. This was further confirmed *in vivo* by instilling another small molecule (EZ-link Sulfo-NHS-Biotin; ThermoFisher, Waltham, MA) in the bladder and assessing its movement into the mucosa. Surprisingly, the area of leakage was not uniform throughout the bladder but localized to very distinct areas while other areas showed no leakage. Furthermore, diabetic mice with NLRP3 deleted did not develop this barrier damage, showing no leakage of dye either *ex vivo* or *in vivo*. This provides unequivocal evidence that inflammation mediated by the NLRP3 inflammasome is a major contributor to the development of urothelial barrier dysfunction during DBD. Mechanistically, there was histological evidence of missing cells creating “holes” in the superficial layer of the diabetic mice with intact NLRP3, suggesting pyroptosis of these cells may be contributing to the leaky barrier. Furthermore, we found significant down regulation of tight junction and adherens junction proteins suggesting a breakdown in these structures (and the regulation of paracellular transport) may also contribute to the loss of barrier function. Such a breakdown exposes the underlying nerves and detrusor to noxious urinary toxins/metabolites which would also contribute to overactivity.

Anticipating that chronic exposure to the triggering agents of DBD would lead to worsening symptoms, we were surprised to find that at a later time point, where UAB symptoms were present, no barrier dysfunction could be found. In addition, expression levels of the tight junction and adherens junction protein were returned to normal. Thus, it appears the urothelial layer has repaired itself by the time the decompensated phase is reached. In this case, where there were no changes apparent, deletion of NLRP3 had no effect. However, it seems likely that NLRP3 prevented the changes from ever occurring at all. Overall, NLRP3 played a major role in the degradation of the barrier function of the urothelia early in the development of DBD.

#### 3.8.6 Smooth muscle dysfunction

Many studies report various alterations in the detrusor during diabetes. For example, STZ induced models have found increased contractility mediated by the release of neurotransmitters and through muscarinic receptors (using carbachol, a muscarinic receptor agonist) (Liu and Daneshgari, 2005; Daneshgari et al., 2006b; Szasz et al., 2016), although most studies have not associated changes in bladder contractility with specific diabetic bladder phenotypes. One exception is in the HFD/STZ model where researchers did find signs of an overactive detrusor that were attributed to increased muscarinic receptor-mediated contractions (Klee et al., 2019). By the time the HFD/STZ rats demonstrated an underactive detrusor, no changes in these contractions were observed (Daneshgari et al., 2006b; Klee et al., 2019; Yesilyurt et al., 2019). Surprisingly, very few studies have directly investigated the presence or the effect of inflammation, much less NLRP3-driven inflammation, on detrusor function during diabetes, although some studies have identified classic pro-inflammatory cytokines and signaling pathways being active. For example, TNFα is increased in the bladder smooth muscle in the DKO model of T2DM and inhibiting TNFα *in vivo* was able to reverse bladder dysfunction (Wang et al., 2012). The single most direct study of the role of NLRP3 employed the db/db model (Priviero et al., 2019). In this model, strips of bladder wall from male mice 14–16 weeks of age show a significant decrease in neurotransmitter-mediated contractions compared to euglycemic controls. Application of the NLRP3 inhibitor MCC950 directly to the strips then restored normal contractions suggesting the NLRP3 activity can rapidly regulate detrusor contractility. Since NLRP3 expression is essentially restricted to the urothelia (Hughes et al., 2015; Hughes et al., 2016a; Hughes et al., 2019a), with little to no expression in the detrusor, it is anticipated that this regulation is secondary through effects from the urothelia.

Arguably, one of the most detrimental effects of diabetes on the detrusor is hypertrophy of the smooth muscle cells and this has been documented in many different models including the STZ model (Liu and Daneshgari, 2006; Kendig et al., 2016), the Akita model (Dolber et al., 2013; Hughes et al., 2022a) and others (Belis et al., 1996; Ellenbroek et al., 2018; Mossa et al., 2020). While diuresis certainly plays a role in causing hypertrophy in the diabetic bladder (Daneshgari et al., 2006a; Fathollahi et al., 2015), hypertrophy is a well-known effect of muscle inflammation. Using a non-diabetic model of bladder inflammation (cyclophosphamide), Halder et al. demonstrated that IL-1β, the output of the inflammasome, directly stimulates bladder smooth muscle expansion. Given the central role we have shown for NLRP3-induced inflammation in DBD, it seems likely that IL-1β is playing a similar role in this disease. Indeed, using the cross breed Akita diabetic/NLRP3^−/−^ mice discussed above, we have recently shown that the detrusor hypertrophy apparent in the Akita mice (female 30 weeks) is prevented in the absence of NLRP3 (Hughes et al., 2022a). Thus, there is considerable evidence that NLRP3-induced inflammation is having significant detrimental effects on the detrusor during diabetes which clearly warrants more in-depth studies.

## 4 Proposed model

**FIGURE 2:**
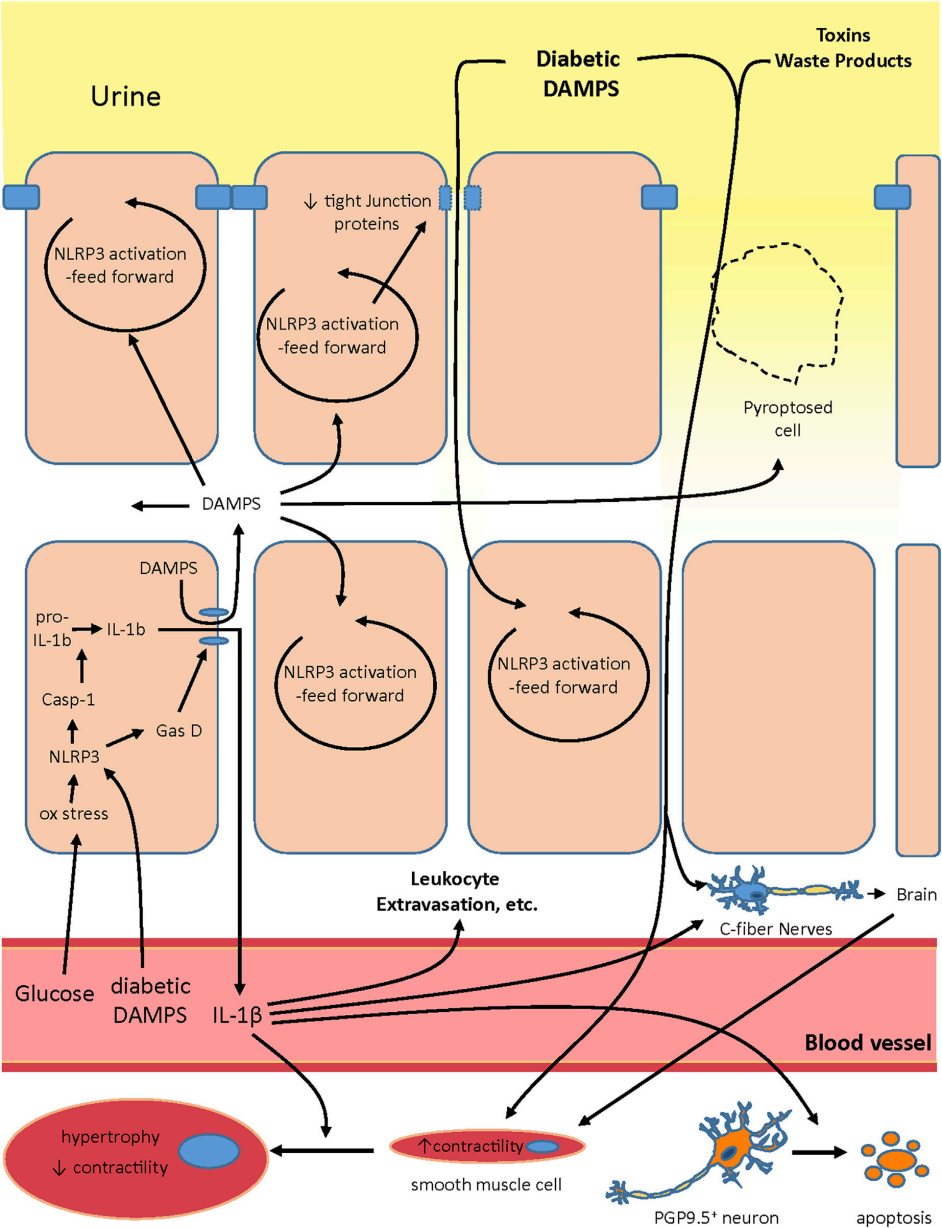
Proposed model of the role NLRP3 plays in creating both the overactive and underactive phase of DBD. Systemic glucose and diabetic DAMPS act in the urothelia to activate NLRP3 and pyroptosis. Pyroptosis then releases intracellular DAMPS that activate NLRP3 in neighboring cells in a feed forward fashion. In the superficial urothelia the feed forward NLRP3 activation also decreases expression of tight junctions. The decrease in tight junctions, along with “holes” in the superficial layer created by pryroptosing cells, decreases the barrier function of the urothelia and allows diabetic DAMPS, toxins and other waste products in the urine to enter deeper into the urothelial layer. These products further activate NLRP3 in nearby cells but also activate nociceptors in C-fibers which relay signals through the brain to the smooth muscles to trigger an increase in contractility, an important component of overactivity. In addition, these noxious compounds could also come in direct contact with smooth muscles and increase their contractility. Systemic DAMPS could contribute to these effects. IL-1β release at the onset of this scenario would act as a pro-inflammatory cytokine and increase leukocyte extravasation and other classical indicies of inflammation. It may also directly activate C-fibers. However, it also seems to drive the progression toward underactivity by directly inducing apoptosis of PGP9.5 + neurons and inducing hypertrophy of smooth muscle cells such that, after the remodeling of the urothelia back into a tight barrier, the bladder in underesponsive to stimuli.

It is intriguing to speculate exactly how NLRP3 plays a role in creating the various changes associated with DBD. Our working hypothesis on the development of OAB during DBD and its progression is illustrated in Figure 2. In the early stages, hyperglycemia creates oxidative stress and this would be particularly prevalent in insulin-independent tissues like urothelia. Oxidative stress is a well-known activator of NLRP3 (Vanaja et al., 2015; Malik and Kanneganti, 2017; Swanson et al., 2019; Yang et al., 2019b; Mamun et al., 2021) and is known to do so in urothelia (Harper et al., 2019). In addition, classic diabetic DAMPs such as MSU, HMGB-1, C6-ceramide, and AGEs would become more prevalent systemically while increasing in concentration in the urine as they are stored for excretion. The systemic diabetic DAMPS would also trigger NLRP3 activation in the urothelia (Hughes et al., 2019a). Activation of NLRP3 would result in gasdermin-D mediated pyroptosis and the release of IL-1β (and possibly IL-18) along with normally sequestered intracellular DAMPS. These released intracellular DAMPS can then activate more NLRP3 in neighboring cells in a vicious feed forward fashion. Activation of NLRP3 in the superficial cells also triggers a reduction in tight junction proteins (Odom et al., 2022), reducing the barrier function of the urothelia. NLRP3-driven pyroptosis of these superficial cells would also create “holes” in the urothelial layers, clearly reducing its barrier function. This compromised barrier would allow the concentrated diabetic DAMPS in the urine greater access to the underlying urothelia, further activating NLRP3 in a feed forward manner. The diabetic DAMPS, toxins and waste products in the exuding urine could then directly activate nociceptors in the C-fibers (Donnelly et al., 2020). Much like the c-fibers, noxious compounds from the extruding urine could come in direct contact with the smooth muscle cells and activate signaling pathways that cause increases in contraction frequency in an effort to rid the bladder of these irritants. Of course, the systemic DAMPS are still present and they further contribute to these effects (not shown in the figure). Also not shown in the figure is the fact that the compromised barrier would significantly facilitate invasion by bacteria from the urine and complicate the inflammatory picture with a urinary tract infection. However, since UTIs are not an essential part of the development of DBD, they are not included in the illustration.

We suspect that IL-1β produced *via* inflammasome activation plays a significant role in creating the overactive phase of DBD. Initially, IL-1β released at the beginning of this sequence would act as a proinflammatory cytokine, triggering changes in the blood vessel wall which increases leukocyte extravasation and other well-known indices of inflammation. NLRP3-produced IL-1β also likely increases the density of c-fibers (Park et al., 2018; Hughes et al., 2019a) which would increase sensitivity to diabetic and intracellular DAMPs and enhance overactivity. Moreover, IL-1β may also directly activate these neurons (Binshtok et al., 2008; Donnelly et al., 2020) triggering signals in the brain for overactivity. It is likely the summed effect of all these factors that brings about diabetic OAB.

As diabetes persists, the inflammation continues and then likely brings about a number of seemingly permanent changes in the bladder, most of which are promoted by IL-1β. One is overall denervation (other than the C-fibers) (Hughes et al., 2019b), which is also likely driven by IL-1β-induced apoptosis of the PGP9.5^+^ neurons (Lutolf et al., 2018), thus reducing the neuronal input in the bladder. IL-1β can also act directly on bladder smooth muscle cells to promote hypertrophy resulting in a hypocontractile state of these cells (Haldar et al., 2015). IL-1β can also drive fibrosis in the bladder (Hughes et al., 2017), although the presence of fibrosis during DBD is controversial at best (Rodrigues et al., 2008; Yankelevitch-Yahav et al., 2015; Wu et al., 2018) and not shown in this model. Eventually the NLRP3-driven damage to the urothelia triggers repair pathways which overcome the NLRP3 input, although the mechanism is not understood, and there is a dramatic remodeling with the barrier becoming tight again (Lutolf et al., 2018). Reforming of the barrier clearly reduces exposure to urinary DAMPS, etc. (although not systemic DAMPS or hyperglycemia) and the organism now experiences UAB symptoms due to the nerve damage and detrusor hypertrophy which are likely permanent or long-lasting. It is interesting that inflammation remains at this stage (Hughes et al., 2022a), likely driven by the systemic hyperglycemia and diabetic DAMPS, yet the feed-forward effects are muted. Future experiments will determine if the inflammatory machinery is reduced in the urothelia at this stage or if there has been an upregulation of anti-inflammatory/pro-resolution pathways (Hughes et al., 2021; Hughes et al., 2022b).

## 5 Conclusion

Over the past 30 years, studies such as DCCT/EDIC have advanced our knowledge of diabetes, its complications, and its treatment considerably. Understandably, these initial investigations focused on the consequences of this disease that carry substantial risk of mortality and morbidity such as vascular and renal disease. From the accrued data, the utilization of strict glycemic control measures in current interventional protocols has led to significant improvement in the management of these patients. With these improvements, attention has shifted to other implications of this disease which may not carry the same risk of disability or death but do have a strong impact on quality of life for a significant number of diabetic patients. Bladder dysfunction has emerged as one of the most common manifestations of diabetes but its nature has been elusive due in part to the complexity of the disease itself but also by concurrent processes, such as BPH in men, or childbirth related incontinence in women, which obscure its etiology and natural course. Future population based trials and longitudinal studies that focus on DBD are ongoing and needed to clarify our understanding of how metabolic dysregulation affects urinary function. Several animal models have been developed and their further characterization will allow us to elucidate the mechanism by which diabetes negatively affects tissues of the lower urinary tract. Combining these avenues of inquiry, a full picture of how DBD develops will continue to emerge.

It is interesting that the UROEDIC study did not find reduction in DBD in response to strict glucose control, even at a 10 years follow up (Van Den Eeden et al., 2009). While the reason underlying this finding is exciting fodder for future exploration, it is not unheard of with other complications and may be the result of what has been termed “metabolic memory” (Ceriello et al., 2009; Copur et al., 2021; Zheng et al., 2021). The most likely explanation for such memory is the nonenzymatic glycation of cellular proteins and lipids, when glucose levels were high. This persists long after these levels are normalized and acts to maintain stress signaling independent of glucose levels. Glycated molecules, particularly Advanced Glycation End Products (AGEs), are diabetic DAMPS well-known to activate NLRP3 and we have shown the bladder is no exception (Hughes et al., 2019a). If such metabolic memory-induced activation of NLRP3 inflammation is driving DBD in the presence of controlled glucose, then targeting the inhibition of NLRP3 may block this pathway and provide relief to patients. Arguably, if the glucose is controlled long-term there might reach a point where the glycated proteins and lipids had all been turned over and NLRP3-inhibition therapy may no longer be needed. However, glycation itself retards protein turnover (Xiang et al., 2001) and some proteins have exceeding low turnover rates (e.g collagen has a half-life of 15 years in skin and 117 years in cartilage (Verzijl et al., 2000) so it may not be surprising, in and of itself, that such memory is still present in the bladder of patients after 10 years of strict glucose control in the UROEDIC study.

While tools available for future study offer promise, we have already made considerable progress and that is most evident in the understanding that diabetic complications result from chronic inflammation induced in the setting of metabolic dysregulation. Diabetic DAMPS activate the NLRP3 inflammasome in tissues including the urothelium of the bladder, as well as the retina, heart, kidney and peripheral nerves. The biology of NLRP3, only discovered at the onset of this century, is also being revealed through intensive study and appears ready to serve as an interventional target to prevent DBD and other organ dysfunction in diabetic patients. Considering that there is currently no specific therapy to treat diabetic urinary dysfunction and that this condition does not respond to strict glycemic control as robustly as other complications, following this line of inquiry could yield important answers in reducing morbidity for the large and growing diabetic community.
